# Sexual effects and long-term outcomes of endoscopic lumbar sympathectomy for plantar hyperhidrosis in men: a cross-sectional study

**DOI:** 10.1590/1677-5449.202400142

**Published:** 2024-09-27

**Authors:** Marcelo de Paula Loureiro, Pietro Maran Novais, Ruimário Machado Coelho, João Augusto Nocera Paulin

**Affiliations:** 1 Universidade Positivo – UP, Curitiba, PR, Brasil.; 2 Universidade Federal do Paraná – UFPR, Hospital de Clínicas – HC, Curitiba, PR, Brasil.; 3 Hospital Nossa Senhora das Graças – HNSG, Curitiba, PR, Brasil.

**Keywords:** endoscopic lumbar sympathectomy, retroperitoneal space, erectile dysfunction, sexual health, male, plantar hyperhidrosis, simpatectomia lombar endoscópica, espaço retroperitoneal, disfunção erétil, saúde sexual, masculino, hiperidrose plantar

## Abstract

**Background:**

Plantar hyperhidrosis (PHH) is a disease with high psychosocial impact, and endoscopic lumbar sympathectomy (ELS) has been shown to be the best choice for treatment, but with some concerns such as compensatory sweating (CS) and sexual effects (SE), particularly in men.

**Objectives:**

The aim of this study is to evaluate the long-term effectiveness of ELS for controlling PHH in men, its side effects, and perceived sexual modifications.

**Methods:**

A cross-sectional study including only male patients operated for PHH with ELS between 2014–2022 at a private practice. During remote interviews, patients were asked about symptoms before and after ELS and about the postoperative effects on PHH. They were also objectively asked about any SE during the postoperative period. Validated quality of life for hyperhidrosis and erectile function questionnaires were also administered.

**Results:**

10 male patients averaging 4.26±2.86 years post-ELS were interviewed. Eight of them (80%) achieved complete response (≥80% of sweat reduction) in the first month after surgery and this response was maintained up to the interview date. Two patients had partial response. In six patients, CS occurred, with 5 reporting it as non-troublesome. Six patients reported some type of SE, but none reported erectile dysfunction. Regarding the functional results, all patients rated ELS from good (10%) to very good (30%) or excellent (60%).

**Conclusions:**

Endoscopic lumbar sympathectomy was effective for treatment of plantar hyperhidrosis in these patients, improving their quality of life and providing lasting PHH control, with some transient sexual dysfunctions that did not impair their sexual life.

## Resumo

**Contexto:**

A hiperidrose plantar (HHP) é uma doença de alto impacto psicossocial, e a simpatectomia lombar endoscópica (SLE) tem se mostrado a melhor escolha de tratamento. Porém, há algumas preocupações como suor compensatório (SC) e efeitos sexuais (ES), particularmente em homens.

**Objetivos:**

O objetivo deste estudo foi avaliar a eficácia a longo prazo da SLE para controlar a HHP em homens, seus efeitos colaterais e as modificações sexuais percebidas.

**Métodos:**

Tratou-se de um estudo transversal incluindo apenas pacientes do sexo masculino operados de HHP por meio de SLE entre 2014 e 2022 em consultório particular. Por meio de entrevistas remotas, os pacientes foram questionados sobre os sintomas antes e depois da SLE e sobre a evolução pós-operatória da HHP, assim como foram questionados objetivamente sobre qualquer ES percebido durante o pós-operatório. Também foram aplicados questionários validados de qualidade de vida para hiperidrose e função erétil.

**Resultados:**

Foram entrevistados 10 pacientes do sexo masculino com média de 4,26±2,86 anos pós-SLE. Oito deles (80%) obtiveram resposta completa (≥80% de redução do suor) no primeiro mês após a cirurgia e mantiveram a resposta na entrevista. Dois pacientes tiveram resposta parcial. Em seis pacientes, ocorreu SC, com cinco relatando-a como não problemática. Seis pacientes relataram algum tipo de ES, mas nenhum apresentou disfunção erétil. Todos os pacientes avaliaram o resultado funcional da SLE como bom (10%), muito bom (30%) ou excelente (60%).

**Conclusões:**

A SLE pode tratar de forma eficaz a HHP nesses pacientes, melhorando a qualidade de vida e proporcionando efeitos duradouros no controle da HHP, com algumas disfunções sexuais transitórias que não prejudicam a vida sexual.

## INTRODUCTION

Plantar hyperhidrosis (PHH) is the excessive production of sweat on the surfaces of the feet due to hyperactivity of the eccrine sweat glands. It usually begins at an early age, between 0 and 19 years of age, but patients generally seek treatment during preadolescence. PHH is sweat that exceeds its physiological function of thermoregulation as to quantity, due to emotional stimuli or stress, and which, in addition to the discomfort of excessive humidity, can lead to strong odors due to fungal infection.^1-3^

Estimates of prevalence in the general population are irregular, ranging from 0.6% to 16.3% and affecting men and women equally. For obvious reasons linked to embarrassment inherent to the disease, prevalence can even be underestimated.^2,4^ Male patients tend to be less concerned about it and fewer men seek treatment for hyperhidrosis (HH), possibly because they are psychosocially less vulnerable to this type of condition than women.^3^

Treatments vary. Initial management is conservative, but the effectiveness of these modalities is very low and all non-surgical treatments have temporary effects. Antiperspirants such as topical aluminum chloride reduce sweating, but can cause skin irritation. Iontophoresis is a treatment option that can be administered at home, but it has side effects.^1,2^ Another option is anticholinergic drugs with systemic effects, but which have not yet been fully investigated for focal HH and have considerable side effects.^1^ Botulinum toxin has been well studied for axillary and palmar HH, but the effect is also temporary, costs are high, and administration if painful.^1,2^ The exception is oxybutynin, which has good long-term effectiveness and is used to indicate surgery, if conservative treatment fails.^5,6^

Endoscopic lumbar sympathectomy (ELS) has been shown to be an effective and long-lasting treatment for PHH. It has still not been adopted as consensus by the International Hyperhidrosis Society because of the theoretical possibility of postoperative complications, although there are publications with great accumulated experience that show the safety of the procedure.^2,3,7-9^ Some prospective studies of ELS for PHH report greater than 90% success.^9-12^

Certain barriers remain to adoption of ELS for PHH in terms of making its results known and enabling it to gain popularity among physicians dedicated to treating this disease. Since there is no universally accepted protocol, it is difficult to access training and become an expert in the technique. In addition, concerns about complications remain, especially compensatory sweating (CS),^1,7-13^ and in the male population it could theoretically have implications for sexual function in the short and medium term.^7-9,14^

The variety of techniques for interrupting sympathetic signals and male anatomical particularities are also challenging, since they potentially influence the possible side effects, mainly erectile dysfunction (ED) and retrograde ejaculation.^8,10,12,14^ Moreover, ELS for PHH is a relatively recent technique, with good medium-term results; but long-term studies and direct investigations of sexual effects (SE) in men are still scarce.^10-12^

## MATERIAL AND METHODS

The present study is a cross-sectional case series with male patients who underwent endoscopic lumbar sympathectomy for treatment of plantar hyperhidrosis at a single center.

Patients were selected from the list of cases treated surgically at a private practice in Curitiba, Paraná, Brazil, from July 2014 to April 2022. The patients recruited were men (over 18 years old) diagnosed with primary plantar hyperhidrosis whose ELS had been conducted at least 12 months earlier. Patients who could not or did not want to answer the questionnaire were excluded from the survey.

During their preoperative interviews, all patients were informed that retrograde ejaculation or ED, and also CS, were possible side effects of the surgery.

The surgical technique used has been described by the authors elsewhere.^8,15,16^

An active search for these patients was conducted by the authors using telephone interviews. Patients were invited to participate in the research and were enrolled after agreeing to take part and digitally signing an informed and consent form.

Data were collected regarding age at surgery, sex, affected regions, HH symptoms, body mass index (BMI), comorbidities (diabetes mellitus), high blood pressure (HBP), neoplasms, thyroid disorders, pituitary disorders, mental disorders, and genetic diseases. Data on continuous use medications were collected from medical charts.

Questions included CS, previous PHH treatments, psychosocial and behavioral changes, impact of ELS on PHH (80% reduction was defined as a complete response and reductions of 50–80% were defined as partial response), level of satisfaction with the results of surgery (from 1 to 10, where 1 is very unsatisfied and 10 is very satisfied), and psychological impact of HH. A translated and validated questionnaire, Quality of Life for Hyperhidrosis (QoLHH), developed by Campos et al.,^17^ was also administered to assess changes in quality of life (QoL) after treatment.

Additionally, questions were objectively asked about SE after surgery and comparatively between before and after surgery. Details were collected about the onset and duration of any preexisting sexual dysfunctions or side effects after ELS, such as erectile changes, penile changes (this was reported spontaneously by the first patient to be interviewed and then included as a direct question thereafter), effects on sexual relations, semen changes, ejaculatory effects, urine color changes, and any other perceived sexual changes.

For patients who reported any type of SE, a second telephone interview was conducted to administer the International Index of Erectile Function (IIEF), developed by Ferraz and Ciconelli,^18^ which classifies erectile dysfunction based on a sum of scores, where 1-10 indicates severe ED, 11-16 moderate ED, 17-21 mild to moderate ED, 22-25 mild ED, and 26-30 represents absence of ED.

In accordance with the Declaration of Helsinki, this study was conducted after approval by the Ethics Committee at the Universidade Positivo, under submission number 61082722.0.0000.0093.

## RESULTS

**Figure 1:**
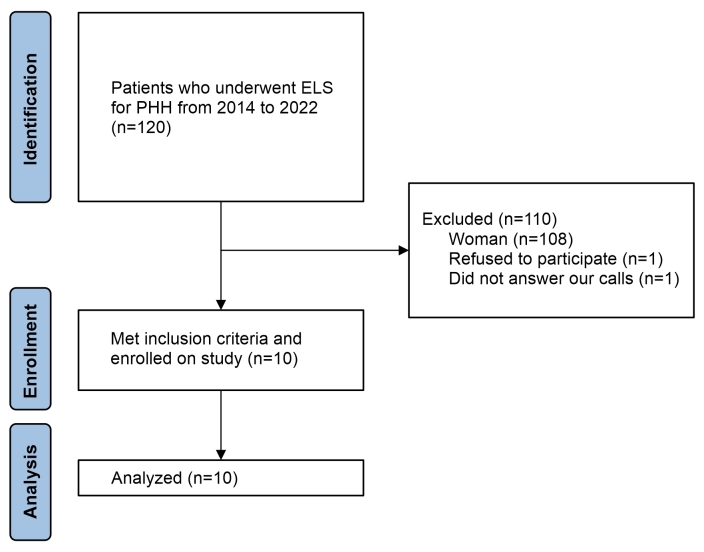
Flowchart of study enrollment. ELS = Endoscopic lumbar sympathectomy. PHH = Plantar hyperhidrosis.

A total of 12 male patients met the inclusion criteria. Two of them were excluded: one did not want to participate and the other did not answer our calls (Figure 1). The average age at operation was 32.8±7.83 years and average time since surgery at interview was 4.26±2.86 years.

Six patients also had foot bromhidrosis, 4 had anxiety when thinking about the condition, 4 reported very cold feet, 2 reported chronic dermatophytosis, and 2 reported chronic onychomycosis.

No intraoperative complications occurred. Specifically, there were no cases of conversion to open procedure, no misidentification of the lumbar ganglia, no peritoneum opening, no lymphatic duct lesions, no lumbar vessel lesions, no need for reoperation or revision, and no deaths.

### Plantar hyperhidrosis control:

In the postoperative follow up, over the first month, 8 out of 10 patients achieved complete response in both feet. Two patients who achieved a partial response still reported a significant reduction in sweat, although less than 80%. Partial recurrence was present in the feet of five patients, with 2 having recurrence at the lateral part of the foot and 2 at interdigital sites; while the fifth patient had recurrence at both regions, with bromhidrosis. Those with recurrence were still maintaining 80% less sweat than before ELS. Compensatory sweating was evaluated, also considering any CS presented after prior endoscopic thoracic sympathectomy (ETS), which had been performed in 7 patients, several years before ELS (Table 1).

**Table 1:** Patient data at baseline, sweat reduction after ELS, presence of CS in patients who had undergone ETS previously, and the effects of ELS on CS.

**Case**	**Age at surgery (years)**	**Previous ETS** ^*^ **(years before our ELS)**	**CS after previous sympathectomy**	**Sweat reduction on both feet (Onset, Duration)**	**New CS areas**	**Post-op time (years)**
1	28	11	No	Partial Response	Yes, Back and Lower Abdomen	8.8
				Immediately, To present		
					After 1 year	
2	29	No	-	Complete Response	Yes, Neck, Upper Chest, and Back	7.7
				Immediately, To present	After 1 year	
3	33	11	Yes, Chest, Back, and Thighs	Complete Response	No	7.2
				Immediately, To present		
4	30	12	Yes, Chest, Bottom, Groin and Feet	Partial Response	No	5.7
				Immediately, To present		
5	45	No	-	Complete Response	Yes, Hands	3.8
				3 Weeks, To present	After 1 year	
6	40	No	-	Complete Response	Yes, Chest and Back	3.5
				3 days, To present	Immediately	
7	25	9	Yes, Back, Lower Abdomen and increased Plantar sweating	Complete Response	Yes, Chest and Thighs	1.9
				7 days, To present		
					After 1 week	
8	43	18	No	Complete Response	Yes, Back	1.6
				Immediately, To present	After 3 weeks	
9	21	5	Yes, Back	Complete Response	No	1.4
				Immediately, To present		
10	34	15	No	Complete Response	No	1.1
				2 days, To present		

ETS = Endoscopic Thoracic Sympathectomy; CS = compensatory sweating; ELS = Endoscopic Lumbar Sympathectomy.

* = Procedures performed by other physicians.

### Other side effects of endoscopic lumbar sympathectomy:

Two patients presented with paresthesia, which was self-limiting. Post sympathectomy neuralgia in the thighs occurred in 2 cases with a verbal numeric rating scale for pain of 8 out of 10, in both cases. One patient was treated with tramadol 50 mg daily for 3 weeks, but pain was self-limiting in the other patient.

Six patients rated the functional result as excellent (9 or 10 out of 10); three patients rated it as very good (7 or 8 out of 10), and one patient rated it as good (6 out of 10). Seven patients reported that they highly recommend the surgery for treating PHH. Patients were also screened for occasional SE after ELS (Table 2).

**Table 2:** Questionnaire results and level of satisfaction with ELS.

**Case**	**QoLHH after ELS**	**Level of satisfaction**	**Sexual effects**
1	A Little Better	7	No
2	A Little Better	8	No
3	Much Better	9	Yes
4	A Little Better	7	No
5	A Little Better	10	Yes
6	Much Better	9	Yes
7	A Little Worse	6	Yes
8	A Little Better	10	Yes
9	A Little Better	10	No
10	Much Better	9	Yes

QoLHH = Quality of life for hyperhidrosis.

### Sexual effects:

Finally, for those patients who reported any perceived sexual effect, a more in-depth assessment of those effects is described in detail (Table 3).

**Table 3:** Evaluation and description of sexual effects.

**Case**	**BMI**	**Changed Libido (Onset, Duration)**	**Penile Changes (Onset, Duration)**	**Sexual Relations Changes (Onset, Duration)**	**Semen Volume Changes (Onset, Duration)**	**Ejaculatory Changes (Onset, Duration)**	**Testicular Changes (Onset, Duration)**	**Use of Antidepressants**	**Erectile function domain (IIEF)**
3	25.5	No	No	No	Yes, Reduction	Yes, Delayed	No	No	29
						1 year, To present			
					1 year, Present				
5	28.73	No	Yes, Enlargement	Yes, Better	No	Yes, Delayed	No	No	30
				6 months, To present		6 months, To present			
			6 months, To present						
6	27.65	No	Yes, Enlargement	No	Yes, Reduction	Yes, Delayed	No	No	30
						4 months, To present			
					Immediately, 18 months				
			7 days, 60 days						
7	24.69	No	No	No	Yes, Reduction	Yes, Delayed	Yes, Reduction	Yes	29
						7 days, 6 months			
								Escitalopram	
							7 days, 4 months		
					7 days, 6 months				
8	30.56	No	Yes, Enlargement	Yes, Difficulty starting	No	Yes, Delayed	No	Yes	29
								Paroxetine Chlorpromazine	
						16 months, To present			
			Immediately, 7 days						
				1 year, 2 months					
10	22.89	Yes, Reduction	No	No	No	No	No	No	29
		Immediately, 2 months							

BMI = Body mass index. IIEF = International Index of Erectile Function.

## DISCUSSION

Lumbar sympathectomy is an intervention in the lumbar sympathetic ganglia between L1 and L5 that has been performed for vascular and neuropathic diseases of the lower limbs since the 20th century. Removal, chemical destruction, or clip control of sympathetic ganglia reduces cholinergic flow (primarily) and adrenergic signal to sweat glands, thus reducing sweat production.^13,19,20^

Use of sympathectomy is already well established for palmar, axillary, or craniofacial HH. For these locations, ETS is the procedure of choice.^21-23^ ETS has been shown to be highly effective in controlling palmar and axillary HH, with 95–96% of patients showing satisfactory control and high levels of approval (92–100%).^22-24^

On the other hand, ELS is still under scrutiny and is not accepted worldwide as the gold standard for controlling PHH, in contrast to its thoracic counterpart. Difficulties such as the learning curve and some potential side effects are used by critics to justify restricting its use.^1,7,8,10-13^

There is very little literature about ELS and significant misunderstanding of the side effects of this technique. Consequently, this does not only affect surgeons, but also, unfortunately, the patients seeking a solution for their suffering. This is especially the case for the male population.^25,26^ Men with PHH are discouraged from undergoing ELS by the majority of surgeons, who simply do not know the effectiveness of the procedure nor the real likelihood of troublesome side effects. Thus, for men, PHH simply does not have any definitive form of treatment, leaving them reliant on local antiperspirants, iontophoresis, or botulinum toxin. However, the available literature shows that the effectiveness observed by different authors is really very high.^8,9,27^

We achieved a 75% rate of total control of PHH, which is slightly inferior to our overall results, published previously.^8,9,14,16^ However, when we consider the effectiveness among the partial response patients, it is clear that there was a substantial level of control of HH in all of the patients’ feet.

Most patients seeking ELS had already had ETS several years previously.^14,28-30^ This is because HH is a disease that affects patients at more than one site simultaneously.^2,4^ It is quite common for patients to have involvement of both hands and feet at the same time. It is consensual to begin treatment with the hands when both surgical procedures are considered, especially because there is a slight potential that plantar HH will be reduced when ETS is performed.^14^ Compensatory sweating is one of the side effects of sympathectomy. It was therefore one of the effects considered in this survey. It is worth mentioning that 60% of our patients had an increase in their CS, which is compatible with what has previously been described for female patients^9^ and a mixed population.^14^ Among those with increased CS, there was one notable case of CS in the hands. That particular patient was a unique case since he had not had ETS prior to ELS and his hands had been uncomfortably dry before ELS. Somehow, after ELS, his hands became wetter, which he considered a very welcome side effect.

Regarding side effects other than sexual dysfunctions, such as post sympathectomy pain, this is very much an expected result. Some patients might need control with pain killers and neurological analgesia for some weeks, but this postoperative pain never becomes chronic.^27,31^

HH is a functional disease; consequently, any evaluation of the effectiveness of treatment is based much more on qualitative analysis. The impact can be better understood by administering questionnaires such as the QolHH, which was developed to assess the impact of treatment on the QoL of HH patients.^17,28^ We still do not have validated questionnaires focused on patients who have undergone ETS and then ELS. As a result, those adaptations could drive some difficult discussions due to analysis of possibly biased questions. Regardless, those were the instruments used to evaluate the QoL and they showed a general improvement after surgery. For us, the most important way to evaluate results is still their level of satisfaction with the surgery, and according to this measure, all the patients classified ELS at least as good and the majority classified it as excellent.

Sexual dysfunctions have always been a focus of concern after lumbar sympathectomy. In particular, studies investigating procedures associated with or due to vascular problems have demonstrated a high incidence of sexual disorders. However, the vascular issues and other factors may have contributed to these results.^11,25,30,32-34^ None of the patients in the present study had ED.

Ejaculation, specifically at the emission phase, is a phenomenon that results from adrenergic stimulation of the sympathetic system. The thoracic sympathetic chain, between levels T10 and L2, innervates the involuntary muscles of the seminal vesicles and the bladder neck.^35^ Thus, providing care is taken to perform sympathectomy below the L3 level, retrograde ejaculation, which was previously a concern, has become an uncommon complication.^27,29,36-38^ None of the patients in this study had this complication.

It is noteworthy that, of the 6 patients who reported some type of sexual change, the most common situations were an increase in ejaculatory latency time (delayed or longer ejaculation) and a (subjective) increase in the size or volume of the penis. In relation to ejaculation, the change was reported by 5 patients, and in 4 it was maintained up to the date of the interview. The increase in penile volume perceived by patients can be easily explained by vasodilation resulting from the reduction in adrenergic tone after sympathectomy and increased blood flow.^13,39,40^

There are no previous reports of delayed ejaculation as a result of lumbar sympathectomy in men. Even after thoracolumbar spinal surgeries, reports are few and the incidence does not exceed 0.1%.^41^ The procedures in the present study were all performed with section of the sympathetic plexus at levels below L2, which reduces the risk of complications related to ejaculation. However, there is evidence that neurons located between L2 and L5 may play an important role in the neurophysiology of ejaculation.^42^

These results may stimulate studies on the physiology of ejaculation and on the role of lumbar sympathectomy in the occasional treatment of ejaculatory disorders in men, as well as evaluation of patients’ erectile function before and after ELS, in addition to any sexual or genital changes, in both sexes,^9,27^ to fully elucidate, primarily, the control of HH and sexual effects. Also, these same findings should be investigated in a larger number of men who have undergone ELS for PHH to corroborate these conclusions. For the moment, ELS should be recommended for all men with PHH refractory to conservative treatment.

## CONCLUSION

Endoscopic lumbar sympathectomy achieved a high degree of control of plantar hyperhidrosis in men, which was maintained over the long term, increased quality of life, did not worsen compensatory sweating, and caused penis enlargement and delayed ejaculation without compromising erectile function or other sexual domains.
